# The Effectiveness and Trade-Offs of Renewable Energy Policies in Achieving the Dual Decarbonization Goals in China: A Dynamic Computable General Equilibrium Analysis

**DOI:** 10.3390/ijerph19116386

**Published:** 2022-05-24

**Authors:** Wei Wei, Ling He, Xiaofan Li, Qi Cui, Hao Chen

**Affiliations:** 1 Management Academy of China Cooperatives, Beijing 100028, China; weiwei19880215@126.com; 2 School of Economics, Beijing Wuzi University, Beijing 101149, China; bjheling@foxmail.com; 3 Beijing Key Lab of Study on Sci-Tech Strategy for Urban Green Development, School of Economics and Resource Management, Beijing Normal University, Beijing 100875, China; 202021410006@mail.bnu.edu.cn

**Keywords:** effectiveness, trade-offs, renewable energy policies, dual decarbonization goals, China

## Abstract

China’s government has enforced a series of renewable energy policies to promote renewable energy development and achieve the dual decarbonization goals. However, there exists great disparity in previous studies on the effectiveness and suitability of renewable energy policies in abating carbon emissions. This study employs a dynamic general equilibrium model and assesses the effectiveness and trade-offs of renewable energy policies in achieving the dual decarbonization goals by 2060 in China. These policies include carbon market (CRP), the reduction of feed-in tariffs (FIT), the reduction of fossil fuel subsidies (FSB), the reduction of renewable energy costs (REC), resource taxes (RTX), and renewable portfolio standards (REP) as well as the mix of these policies. We find that renewable energy policies together could abate China’s CO_2_ emissions in 2060 by 2.57 billion tons, but their effectiveness is very different. The REC would have the greatest effectiveness in abating CO_2_ emissions, followed by REP and CRP. Renewable energy policies would cause relatively slight damage to China’s GDP, with the exception of the REC (raising GDP by 1.1713%). Regarding trade-offs, most policies will sacrifice China’s internal and external demand but benefit employment. Renewable energy policies will effectively promote the low-carbon transformation of China’s energy structure.

## 1. Introduction

Achieving the national goals of “dual decarbonization” is not only essential for China’s future sustainable development by 2050, but also contributes substantially to global carbon emission reduction. In 2019, China’s CO_2_ emissions reached 99.19 million tons, accounting for 29.5% of the world’s total CO_2_ emissions [1]. As the largest carbon emitter, China announced the dual decarbonization goals in 2020, stating that “the country will peak carbon emissions before 2030 and achieve the carbon neutrality by 2060”. The attainment of these decarbonization goals, although promising, may require a pronounced decrease in fossil fuel consumption and the transformation to a low-carbon energy system, which heavily depends on the development of renewable energy [2,3,4]. All the studies that evaluated China’s dual decarbonization scheme regarded the substitution of renewable energy for fossil fuels as the most important measure to achieve dual decarbonization goals [5,6,7,8,9]. Zhang and Huang et al. (2022) also projected that the proportion of renewable energy in primary energy consumption must surpass 65% by 2060 to achieve the carbon neutrality goal [8]. Hence, the attainment of China’s dual decarbonization goals requires the rapid development of renewable energy and the low-carbon transformation of the energy system.

**Figure 1:**
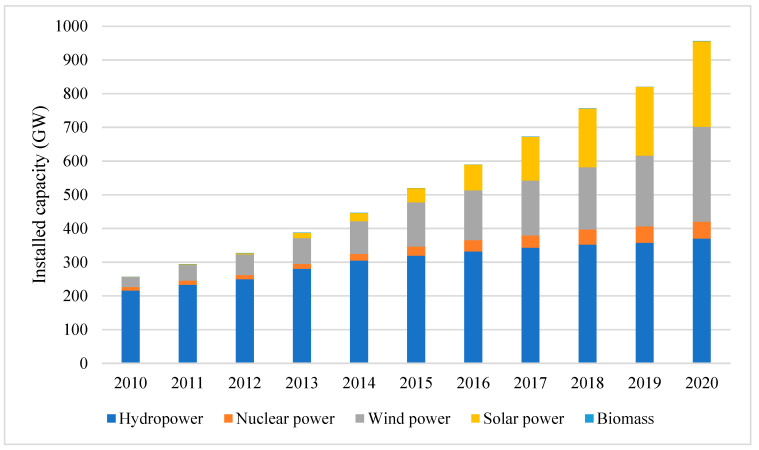
The installed capacity of renewable energy powers in 2010–2020. Source: China Statistical Yearbook, 2021.

In the past decade, China has enforced a series of supportive policies to accelerate the development of renewable energy. These policies were mostly market-oriented and aimed to raise the comparative competitiveness of renewable energy against fossil fuels and fossil-fired electricity, including feed-in tariffs, tradable green credits, renewable portfolio standards, electricity market reform, and carbon trading markets as well as policies supporting the research and development of renewable energy technologies [10,11,12,13,14,15]. The Modern Energy System Planning in the 14th Five Year Plan released in early 2022 announced that non-fossil energy will account for 39% of total power generation and 20% of primary energy consumption by 2025. The document also required that renewable energy become the main body of China’s power source by 2035. Benefiting from these policies, the installed capacity and power generation of renewable energy have been growing rapidly since 2010. The installed capacity of renewable energy reached 955.79 GW in 2020, almost three times larger than that in 2010 (256.75 GW, Figure 1). The power generation of renewable energy has increased from 0.80 PWh in 2010 to 2.20 PWh in 2020, with an annual average growth rate of 10.63%. However, by 2020, renewable energy accounted for only 28.23% of total power generation and 13.32% of primary energy consumption in China [1]. Given the ambitious dual decarbonization goals, more aggressive policies should be implemented to accelerate the development of renewable energy in the coming decades.

A large number of studies have evaluated the effectiveness of renewable energy abating CO_2_ emissions, but they have great disparities. Most studies believe that the development of renewable energy will effectively mitigate China’s carbon emissions by substituting fossil fuels and fossil-fired electricity [16,17,18]. They also found that renewable energy policies could play a vital role in achieving China’s carbon peak goal before 2030, such as carbon market [19,20], reduction in fossil-fuel subsidies [21,22,23], electricity market reform [17,24,25], renewable energy portfolio standards [26,27], and mix of other policies [28,29]. Nevertheless, several recent studies have found that current renewable energy policies are not able to abate CO_2_ emissions effectively [30,31,32]. For example, Guo et al. (2021) found that the improvement of renewable energy technologies could not prevent carbon emissions from continuously increasing toward 2030, and the carbon peak goal may not be realized [30]. Cao et al. (2021) also suggested that the single policy of the carbon market could not achieve the carbon neutrality goal, and more ambitious policies are needed, such as rapid electrification and deep economic transformation [31]. Furthermore, Zhao and Zhong et al. (2022) even found that the incremental consumption of renewable energy would increase carbon emissions, as the development of renewable energy may crowd out nuclear power and increase the peak-shaving demand for fossil-fired electricity [32]. Furthermore, to our knowledge, previous studies have not explicitly examined and compared the effectiveness of different renewable energy policies in achieving China’s carbon neutrality goals.

Existing studies also have great disparities in the economic impacts of different renewable energy policies. Several studies confirmed that these policies would cause economic losses to a certain degree, by reducing GDP, employment, and sectoral output, reflecting the costs of carbon emission abatement [33,34,35,36]. However, many studies have found positive effects associated with renewable energy policies on economic growth [8,20,37]. The development of renewable energy would increase the investment, create new jobs, and stimulate the technical innovations, consequently promoting the long-term economic growth [5,38,39]. More complicatedly, renewable energy policies would have very different impacts on various elements of the economy, such as employment, investment, consumption, and export [24,40,41]. Some policies may improve investment and consumption but worsen exports [42,43], indicating the trade-offs underlying these policies that were not explored by previous studies. Therefore, the economic effects of renewable energy policies for achieving China’s dual decarbonization goals should be investigated comprehensively, through revealing their economic costs and trade-offs.

This study employs a dynamic general equilibrium model and assesses the effectiveness and trade-offs of renewable energy policies in achieving the dual decarbonization goals in China by examining the multidimensional impacts on carbon emissions, economic growth, and sectoral output. We establish a baseline scenario for China’s economic growth and carbon emissions toward 2060, and seven scenarios for renewable energy policies. These policies include the carbon market, the reduction in feed-in tariffs, the reduction in fossil fuel subsidies, the reduction in renewable energy costs, resource taxes, and renewable energy portfolio standards as well as the mix of these policies. This study contributes to the literature in the following ways. First, the effectiveness of different renewable energy policies in abating China’s carbon emissions toward 2060 are quantitively evaluated. Second, the trade-offs of renewable energy policies are investigated by comparing the changes in different economic indicators, which reveals the costs of these policies more comprehensively. Third, an efficiency index for different renewable energy policies is created to describe the suitability of these policies in achieving China’s dual decarbonization goals. In addition, this study will provide several insightful implications for making renewable energy policies under the dual decarbonization scheme.

The remainder of this paper is organized as follows. Section 2 introduces the simulation model, data, and scenarios. Section 3 shows the simulation results for the impacts of renewable energy policies on CO_2_ emissions and economic indicators, revealing the effectiveness and trade-offs of the policies. Section 4 discusses the implications and limitations of the results. The last section concludes this study.

## 2. Methodology

### 2.1. CHINAGEM Model

The CHINAGEM model is a multi-sector dynamic CGE model, developed by the Institute of Science and Technology, Chinese Academy of Sciences, and the Center of Policy Study, Victoria University. As the CGE model can capture the direct and indirect effects of exogenous changes in the economy and identify the impact mechanisms across the economy, it provides a useful tool for a variety of policy-oriented studies related to macroeconomic, trade, and environmental policies. The CHINAGEM model is widely used in analyzing the effects of energy policies [31,42,44]. Based on neoclassical economic theory, it assumes that the market is fully competitive, and the returns to scale of production remain unchanged. The modules of production, investment, household coal consumption, export, equilibrium, and closure are briefly introduced in Cui et al. (2020) [42]. To save space, we only describe the improvement in the production module and the CO_2_ emissions from the production process.

**Figure 2:**
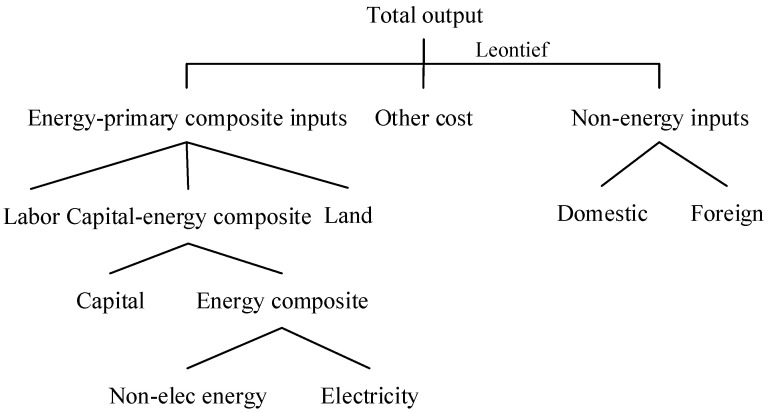
The nesting structure of the producing input used by sectors.

**Figure 3:**
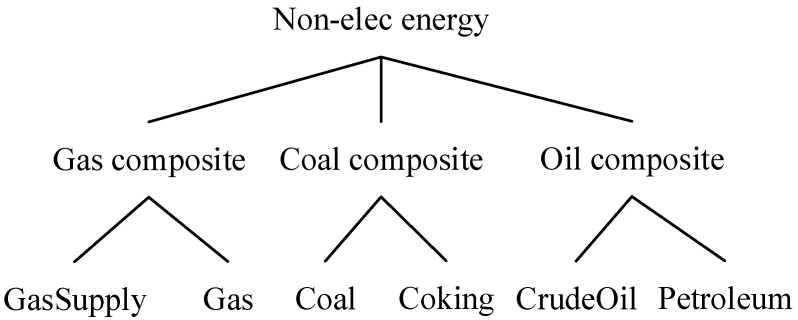
The nesting structure of non-electricity energy used by producing sectors.

In the production module, the nested constant elasticity of substitution (CES) functions are used to describe the substitution of different energy commodities consumed by each production sector. The substitution elasticities for different production input and energy products are from Cui et al. (2020) [42]. On the top level of Figure 2, the utilizations of intermediate inputs, primary factors, and other costs are fixed proportional to each sector’s output described by the Leontief function, which is a special CES function with the substitution elasticity of 0. The primary factor is composited by land, labor, and capital-energy composited product, with the substitution elasticity of 0.5. Then, capital is regarded as a partial substitution for the energy product, as firms can utilize highly efficient machineries to save energy input when facing increasing energy prices. The substitution elasticity between capital and energy products is set to 0.5. On the next level, the energy product is bundled by electricity and non-electricity products. The electricity and non-electricity products are partially substituted, described by a CES function with the substitution elasticity of 0.5. As shown in Figure 3, the non-electricity product is composed of coal, oil, and gas, described by a CES function with the substitution elasticity of 0.16. On the bottom level of non-electricity products, coal is composited by crude coal and coke, oil is composited by crude oil and petroleum products, and gas is composted by crude gas and gas supply. The substitution elasticities at this level are set to 0.5.

**Figure 4:**
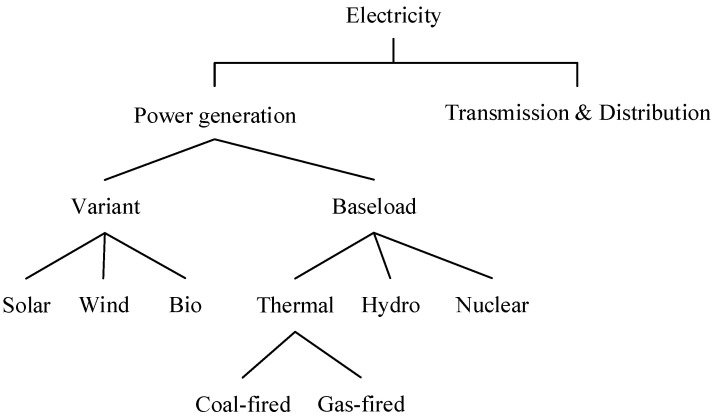
The nesting structure of electric powers used by producing sectors.

Compared with non-electricity products, the structure of electricity with different power sources is much more complicated (Figure 4). The CHINAGEM model incorporates seven electricity generation sectors with different power sources, including coal-fired, gas-fired, nuclear, hydro, wind, solar, and biomass power, and one sector for electricity transmission and distribution. At the top of the nesting structure of electricity, the Leontief function is employed to assume the utilization of electricity as a fixed proportion of transmission and distribution. Then, electricity utilization is composited by the base-load and variant-load power, described by a CES function with the substitution elasticity of 3. Then, the base-load power is composited by the thermal power, hydropower, and nuclear power, with the substitution elasticity of 5. The variant-load power is composed of solar power, wind power, and biomass power, with the substitution elasticity of 5. At the bottom level, thermal power is composed of coal-fired and gas-fired power, with the substitution elasticity of 10.

Based on the improved production module, we establish the module for calculating CO_2_ emissions from the combustion of fossil fuels and the production process. For the former, CO_2_ emissions are calculated by multiplying CO_2_ emission coefficients with the combusting quantity of fossil fuels. For the latter, the CO_2_ emissions from the production process are directly correlated with the output of cement. Therefore, we regard it as an unintended production input of cement, which is valued at the carbon price. Hence, the production cost of cement is composited by the total input and the value of CO_2_ emissions. We assume that the total input and the value of CO_2_ emissions are partially substituted, as the firm would reduce the production input and output of cement when facing the rising carbon price. The substitution is described by the CES function with the substitution elasticity of 0.5.

Renewable energy policies would have very different impacts on GDP and CO_2_ emissions. The evaluation of renewable energy policies should compare the impacts on the GDP and CO_2_ emissions simultaneously. Therefore, we calculate a simple indicator to measure the efficiency of renewable energy policies in abating CO_2_ emissions through dividing the percentage changes in China’s GDP by the amount of carbon emission abatement (Equation (1)). This indicator gauges the average economic loss for abating per billion tons CO_2_ by renewable energy policies.

(1) $$EI=\frac{\Delta GDP/GDP\times 100}{\Delta CO_{2}}$$

where ΔGDP and $\Delta CO_{2}$ are the changes in China’s GDP and carbon emission in absolute values, respectively. The positive value suggests that the policy would benefit the economy while abating CO_2_ emissions, vice versa. Hence, a higher value of the indicator indicates a more efficient policy in abating carbon emissions.

### 2.2. Data

To construct a database for the CHINAGEM model, we make use of China’s 2017 input–output table with 149 original production sectors. As there is only one electricity sector in the original input–output table, the original electricity sector is split into nine new sectors with different power sources, including eight generation sectors (coal-fired, gas-fired, nuclear, hydro, solar, onshore wind, offshore wind, and biomass and geothermal power) and one sector of power transmission and distribution. To split the electricity and gas sector, we follow the methodology developed by Wing (2008) [45]. The essence of the procedure is to minimize the deviations between the benchmark allocation of inputs to the discrete technologies and the input cost shares implied by their engineering characteristics, subject to the zero-profit and market-clearance constraints of the production structure of different power sectors. We employ the data on the input coefficient of electricity sectors provided by the China database of the GTAP-E-Power model [46] and the data on electricity generation of power sources from the China Electric Power Yearbook (2018). Similarly, the original crude oil and gas sector is split into three new sectors: crude oil, pipeline gas, and LNG. Thus, we end up with 159 production sectors. The elasticities of the demand and supply equations used in the CHINAGEM model are adopted from Cui et al. (2020) and Cui et al. (2022) [42,47].

The CO_2_ emissions from the fossil fuel combustion and cement production process are incorporated into the CHINAGEM model. CO_2_ emissions from burning fossil fuel are calculated by multiplying sectors’ and households’ fossil fuel consumption with the corresponding carbon emission factors. The carbon emission factors of fossil fuels, including coal, crude oil, natural gas, coke, petroleum products, and gas products, are sourced from the National Development and Reform Commission (2011). CO_2_ emissions from cement production process are derived from Shan et al. (2019) [48]. They suggested that China’s CO_2_ emissions from the process of cement production process in 2017 reached 700.44 million tons. We do not consider CO_2_ emissions from the production process of other sectors. On one hand, cement accounted for over 70% of China’s CO2 emissions from the production process. On the other hand, we could not obtain the reliable data on CO2 emissions of other sectors. Hence, this study only considers CO_2_ emissions from cement production with the data that are provided by Shan et al. (2019).

### 2.3. Scenario Design

To simulate the effects of renewable energy policies in achieving China’s dual decarbonization targets, a baseline scenario for 2060 is established to reflect the changing trends of economic development and energy transformation under current energy policies. The energy consumption and CO_2_ emissions in China during the period 2017–2050 are calibrated to the projections by the International Energy Agency [49]. The energy consumption and CO_2_ emissions in 2050–2060 are extrapolated by the change rates in the period of 2040–2050.

The CO_2_ emissions from fossil fuel combustion reached a level of 9.27 billion tons in 2017, and are projected to peak in 2028 at 10.47 billion tons. Then, CO_2_ emissions from fossil fuel combustion will fall persistently and decrease to 8.09 billion tons by 2060. The CO_2_ emissions from cement production are projected to peak in 2024 at 0.73 billion tons, and then decline continuously, accompanied by the extraction of cement production. The CO_2_ emissions from cement production will decrease to 0.20 billion tons by 2060. By adding the CO_2_ emissions of fossil fuel combustion and cement production, China’s total CO_2_ emissions will peak in 2028 at 11.20 billion tons, and fall to 8.29 billion tons by 2060. Primary energy consumption is projected to increase from 4.47 billion tce (ton of standard coal equivalent) in 2017 to 6.30 billion tce in 2060, with an annual average growth rate of 0.84%. The proportion of renewable energy will rise from 11.54% in 2017 to 36.08% in 2060.

We design seven scenarios concerning different types of renewable energy policies and the implementation of the carbon market. The renewable energy policies studied here include the reduction in feed-in tariffs, the reduction in fossil-fuel subsidies, the reduction in renewable energy costs, the increase in resource taxes, and the increase in the proportion of renewable energy (summarized in Table A1). These policies have been included in climate and energy policies enforced by China’s government. However, the strength of these policies designated by this study is higher than the current level, as China’s government must implement more stringent policies to achieve the dual decarbonization goals. (1) The carbon market (CPR) will raise the carbon price in China from 100 RMB/ton CO_2_ in 2020 to 100 USD/ton CO_2_ in 2060, which is near the annual averaged increase of 5%. The price of carbon trading system has reached a level of 42.85 RMB/ton CO_2_ in 2021 (around 6 USD/ton CO_2_). (2) The reduction in feed-in tariffs (FITs) would cancel levying feed-in tariffs on fossil-fuel fired powers and subsidizing renewable energy from 2020. China’s government tended to replace the feed-in tariff with the green-certificate trading system in the subsequent years, to accommodate renewable energy at the equal price to fossil-fired electricity. In 2020, the revenue of feed-in tariffs collected from fossil fuel-fired powers reached 88.4 billion RMB, and the subsidy on renewable energy was 92.4 billion RMB. (3) The reduction in fossil fuel subsidies (FBS) will reduce governmental subsidies on fossil fuel from 2020. In 2019, the subsidy for petroleum products was 18.1 billion USD and that for fossil fuel-fired power was 12.4 billion USD. (4) The reduction in renewable energy cost (REC) considers the continuous decrease in the generation costs of renewable power. As suggested by Energy Intelligence (2020), the generation costs of renewable power would continuously decline during the period of 2020–2050, with annual averaged decreasing rates from 0.505% to 2.954%. We assume that the generation costs of renewable power will maintain a decreasing trend in 2050–2060. (5) The policy of resource tax (RTX) will raise the rate of resource taxes for coal, crude oil, and crude gas from the current level of 6% to 7.5% in 2020. (6) The renewable energy portfolio standard (REP) will increase the proportion of renewable energy in primary energy consumption by an additional 5 percent from 2020 to 2060. In the past years, China’s government raised the proportion of renewable energy persistently through the accommodation of curtailed renewable energy, electricity market reform, and infrastructure construction. As declared by an official document of China’s government, the proportion of non-renewable energy will reach a level of 25% by 2030. (7) The mix of renewable energy policies (MIX) will implement the aforementioned policies simultaneously. By comparing the simulation results among different scenarios, we reveal the effectiveness and trade-offs of different renewable energy policies in achieving dual decarbonization targets in China.

## 3. Results

In this section, we discuss the simulation results for the effects of renewable energy policies on carbon emissions, the macroeconomy, and energy structure and reveal the effectiveness and trade-offs of these policies. Then, we calculate an indicator measuring the efficiency of carbon emission abatement for different policies.

### 3.1. The Effectiveness of Renewable Energy Policies in Abating CO_2_ Emissions

**Figure 5:**
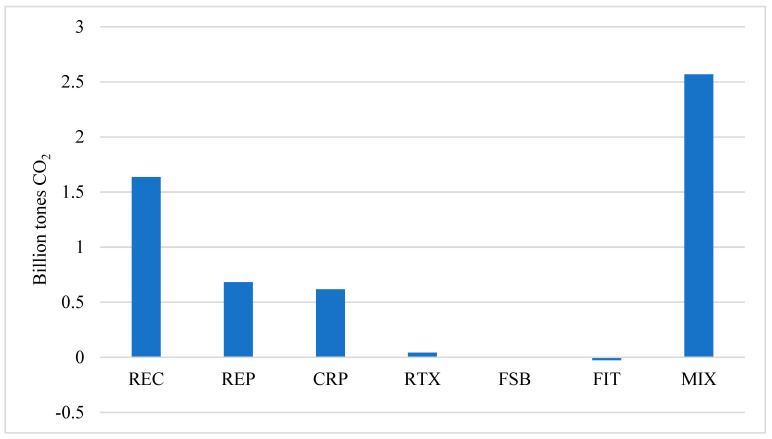
The effectiveness of renewable energy policies in abating CO_2_ emissions in 2060. Source: CHINAGEM model simulation.

Except for reducing the feed-in tariff (FIT), renewable energy policies could reduce China’s CO_2_ emissions toward 2060, but their effectiveness in abating CO_2_ emissions is very different (Figure 5). Among them, the reduction in renewable energy cost (REC) would have the greatest effectiveness in abating CO_2_ emissions toward 2060, reducing 1.636 billion tons CO_2_ in 2060, which accounts for 19.52% of China’s carbon emissions in 2060 under the baseline scenario. This policy will continuously reduce the generation costs of renewable energy and enhance the competitiveness of renewable powers, stimulating firms and households to substitute fossil fuels and fossil-fired electricity with renewable electricity. Following the RCE, renewable energy portfolio standard (REP) and carbon market policies (CRP) would reduce CO_2_ emissions by 0.682 and 0.617 billion tons, respectively, in 2060. The REP would increase the proportion of renewable powers in primary energy consumption to replace only fossil-fired electricity, which is different from the REC. The CRP would not only increase the burning cost of fossil fuels utilized by firms and households, reducing CO_2_ emissions from the combustion of fossil fuels but also reduce the output of cement and CO_2_ emissions from the production process.

Comparably, the increase in resource tax (RTX) and reduction in fossil-fuel subsidy (FSB) would have the relatively smaller abatement of CO_2_ emissions. They will mitigate CO_2_ emissions in 2060 by 0.043 and 0.005 million tons, respectively. By raising resource taxes on fossil fuels and reducing the subsidy on fossil fuels, these policies could increase the combustion cost of fossil fuels relative to renewable energy, which consequently stimulates firms and households to substitute fossil fuels with renewable energy. However, both resource taxes and subsidies accounted for a much smaller proportion in the cost of fossil fuel. Hence, the effectiveness of RTX and FSB is much lower than that of REC, REP, and CRP. In contrast to the aforementioned policies, the reduction in feed-in tariff (FIT) would raise CO_2_ emissions in 2060 by 0.03 billion tons, as it will cut down the subsidy on renewable energy. In addition, if renewable policies are implemented together (MIX), CO_2_ emissions in 2060 would reduce by 2.57 billion tons, accounting for 30.59% of China’s carbon emissions under the baseline scenario. Therefore, renewable energy policies could play a key role in achieving the dual decarbonization targets, as they will mitigate over 30% of CO_2_ emissions by 2060.

### 3.2. The Abatement Cost of Renewable Energy Policies

The abatement cost of renewable energy policies is measured by the changes in China’s GDP. We find that renewable energy policies would cause relatively slight damage to China’s GDP, with the exception of the REC (Table 1). Among these policies, the CRP policy would cause the largest economic loss, by reducing China’s GDP by an accumulative 0.0989% in 2020–2060. The CRP policy would raise the combustion cost of fossil fuels paid by firms and households and reduce industrial production and household consumption, which consequently lowers the GDP. Meanwhile, the revenues of the carbon market will be recycled to the production sectors, alleviating the economic losses caused by the increasing carbon price. Hence, the carbon price that will increase to 100 USD/ton CO_2_ in 2060 would only cause slight damage to China’s GDP. Following the CRP policy, the REP and RTX policies would reduce China’s GDP by accumulatively 0.0384% and 0.0144% in 2020–2060, respectively. The REP policy would raise the production of renewable energy in primary energy consumption, but largely reduce the production of fossil fuels and fossil-fired electricity. Weighting the contradictory impacts, the REP policy would cause slight damage to China’s GDP. The RTX policy could bring welfare loss, similar to the typical tax, as it distorts the efficient allocation of resources guided by the market price. Compared with these policies, the negative impacts of the FSB and FIT policies are much softer, reducing the GDP by accumulatively 0.0023% and 0.0019%, respectively, in 2020–2060. It is worth noting that the REC policy could significantly benefit economic growth by raising China’s GDP by accumulatively 1.1713% in 2020–2060. Even considering the cost of improving renewable energy technology, the REC policy will lower the electricity price and stimulate industrial production, which raises the growth rate of GDP. If these energy policies are enforced together (MIX), China’s GDP will increase by accumulatively 1.1811% in 2020–2060, which is driven by the positive impact of the REC.

### 3.3. The Trade-Offs of Renewable Energy Policies

As renewable energy policies would have very different impacts on various elements of China’s macroeconomy, the analysis of the trade-offs underlying the impacts of renewable energy policies on the macroeconomic indicators could reveal the costs of different policies more comprehensively (Table 1).

As for China’s internal demand, most renewable energy policies will sacrifice investment and consumption, but benefit employment. Except for the FIT, renewable energy policies would raise employment by a range of over 0.0009% to 0.0936% in 2020–2060, as renewable energy policies would expand the production of renewable energy sectors and increase labor demand. If renewable energy policies are implemented together, China’s employment in 2060 will increase by 0.1720%. The positive effects on employment could be regarded as the economic benefit of renewable energy policies. Meanwhile, renewable energy policies will also reduce the production of fossil fuels and fossil-fired electricity and reduce capital demand, as these industries are mostly capital-intensive. Except for the REC and REP policy, the investment will decline by a range over 0.0011% to 0.0533% in 2020–2060. However, the REC and REP could increase investment in renewable energy sectors, exceeding the decline of the investment in non-renewable energy sectors, which consequently benefits China’s total investment. Except for the REC policy, renewable energy policies will reduce consumption by over 0.0011% to 0.0638% in 2020–2060. In contrast, the REC policy will raise the consumption by over 1.1704% in 2020–2060. Therefore, most renewable energy policies will abate CO_2_ emissions at the cost of decreasing China’s internal demand (i.e., investment and consumption) but improve employment. Only the REC policy could promote investment, consumption, and employment simultaneously.

In addition to the negative impact on China’s internal demand, the renewable energy policies would also damage the external demand, except for the REC policy. These policies will reduce exports by a range of over 0.0007% to −0.1121% in 2020–2060, as they raise the costs of fossil fuel and fossil-fired electricity and force firms to use more expensive renewable energy, weakening the competition for China’s exports in the global market. The only exception is the REC policy, which will increase exports by 0.9507% in 2020–2060, because this policy could lower the electricity price and industries’ production costs in China. Hence, the REC policy will benefit China’s both internal and external demand, while abating CO_2_ emissions most effectively. Owing to the positive impact of the REC policy, the mix of renewable energy policies will improve China’s investment, consumption, employment and exports.

### 3.4. The Carbon Reduction Efficiency for Renewable Energy Policies

As discussed in Section 3.1 and Section 3.2, renewable energy policies would have very different impacts on GDP and CO_2_ emissions. While several policies abate CO_2_ carbon emissions more effectively, such as the REP and CRP policies, they cause greater GDP losses. The indicator calculated by Equation (1) would evaluate the carbon reduction efficiency of renewable energy policies.

**Figure 6:**
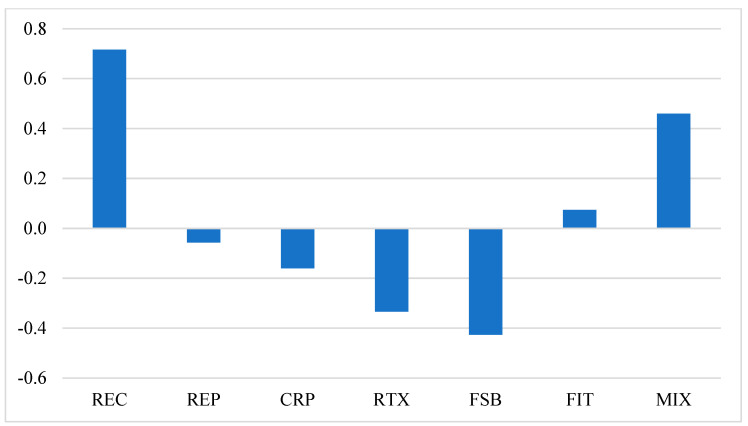
Carbon reduction efficiency for renewable energy policies.

According to the efficiency indicators in Figure 6, the renewable energy policies could be categorized into three groups. (1) The REC and FIT policies have positive efficiency indicators, but the results have very different meanings for two policies. The positive indicator (0.716) of REC policy indicates that it will benefit the economy while abating CO_2_ emissions. To abate CO_2_ per billion tons, the REC policy will increase China’s GDP by 0.716%. However, the positive indicator of FIT policy suggests that the cancel of feed-in tariff will increase CO_2_ emissions and damage the GDP. The maintenance of feed-in tariffs on fossil-fired electricity to subsidize renewable electricity could simultaneously reduce CO_2_ emissions and improve economic growth. (2) Although the efficiency indicators for the REP and CRP policies are negative, their efficiency is much higher than that for the RTX and FSB policies. The efficiency indicators of the REP and CRP policies are 0.056 and 0.160, respectively, which suggests that for abating per billion tons CO_2_, these policies will cut down China’s GDP by less than 0.2%. Although these policies would cause relatively great damage to the GDP, they have much higher efficiency in abating CO_2_ emissions in terms of the average GDP loss for abating per billion tons CO_2_, compared with RTX and FSB policies. (3) While the RTX and FSB policy will cause relatively slight damage to the GDP, their efficiency in abating CO_2_ emissions is much lower. The efficiency indicators of the RTX and FSB policies are 0.334 and 0.427, respectively, which suggests that for abating per billion tons CO_2_, these policies will reduce China’s GDP by more than 0.3%. In addition, if the renewable energy policies are implemented simultaneously, the efficiency indicator of the MIX policy reaches 0.460, indicating that to abate CO_2_ per billion tons, China’s GDP will increase by more than 0.4%. Therefore, by ranking renewable energy policies according to the efficiency indicator, the REC policy is the most suitable policy to promote the development of renewable energy, followed by the REP and CRP. The RTX and FSB policies are of the least suitability for achieving China’s dual decarbonization goals.

### 3.5. The Impacts of Renewable Energy Policies on Energy Production

**Figure 7:**
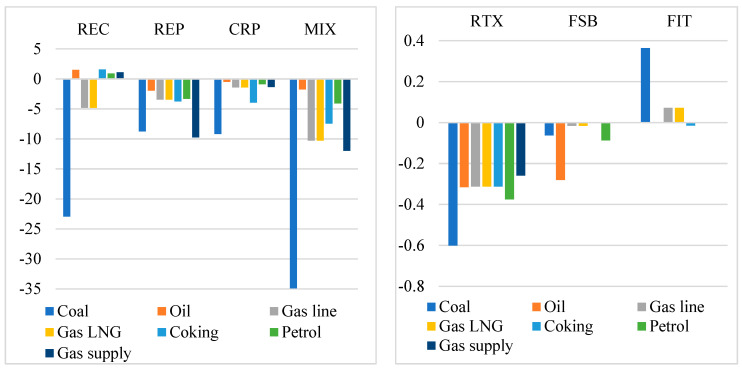
The accumulative changes in the output of fossil fuels in 2020–2060 relative to the baseline scenario (%). Source: CHINAGEM model simulation.

Renewable energy policies will deeply reshape the structure of energy production in China and foster the low-carbon transformation, by reducing the production of fossil fuels and raising the proportion of renewable energy. Most renewable energy policies will significantly reduce the output of fossil fuels, as these policies will replace fossil fuel and fossil-fired electricity with renewable energy (Figure 7). This also could be regarded as the other benefit brought by renewable energy policies. Among the single policies, the REC, REP, and CRP policies will pose relatively greater negative impacts on the output of fossil fuel. For example, they will reduce the output of coal by 22.93%, 8.23%, and 9.17% in 2020–2060, respectively, relative to the baseline scenario. Comparably, the RTX and FSB policies will only reduce the output of coal by 0.60% and 0.06%, respectively. Compared with coal, the reductions in crude oil, crude gas, and energy products (coke, petroleum product, and gas product) are relatively smaller. However, the REC policy will increase the output of crude oil and energy products, as it will stimulate industrial production and increase investment and export, which leads to an increase in crude oil and energy products. In addition, the FIT policy will slightly increase the output of fossil fuels. If renewable energy policies are implemented simultaneously, the output of coal, crude oil, and crude gas will fall by 34.92%, 1.76%, and 10.29%, respectively.

**Figure 8:**
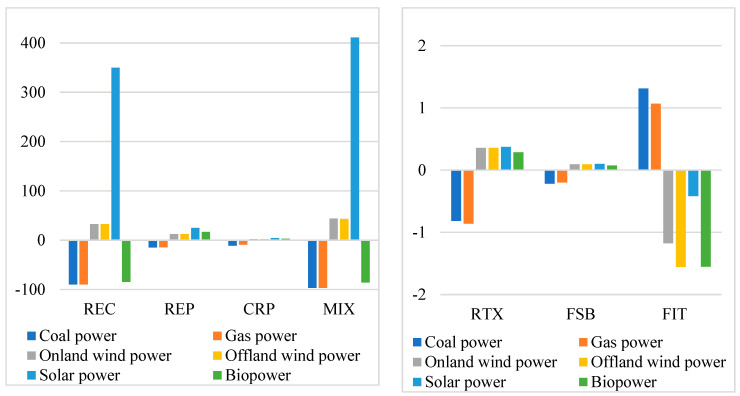
The accumulative changes in the output of electricity in 2020–2060, relative to the baseline scenario (%). Source: CHINAGEM model simulation.

Renewable energy policies will increase the proportion of renewable energy in electricity generation significantly, as substitutes for fossil fuel and fossil-fired electricity will be substituted with renewable energy (Figure 8). Among the renewable energy, the output of solar power will increase most significantly, followed by wind power. If the renewable energy policies are implemented together (the MIX policy), the output of solar power will increase by over 400% in 2020–2060 relative to the baseline scenario. The output of wind power will also increase by over 40% from the period of 2020–2060. Simultaneously, the output of coal-fired power and gas-fired power will fall by over 95%, and that of biomass power will decline by over 85%. The change in electricity generation structure is dominantly driven by the REC policy. Determined by the nested CES structure for electric powers, the continuous decrease in the generation cost of renewable energy will force the firms to substitute non-renewable electricity with renewable electricity. The solar power has the most rapid decrease in the generation cost, giving it the comparative advantage to other powers. As a result, the REC would largely raise the proportion of solar power and reduce the output of fossil-fired powers. In addition, compared with coal-fired electricity, renewable energy policies will have a relatively smaller impact on coal production, which is also determined by the nested CES structure for energy products. Therefore, the current policies could effectively substitute for fossil-fired electricity rather than fossil fuels.

**Figure 9:**
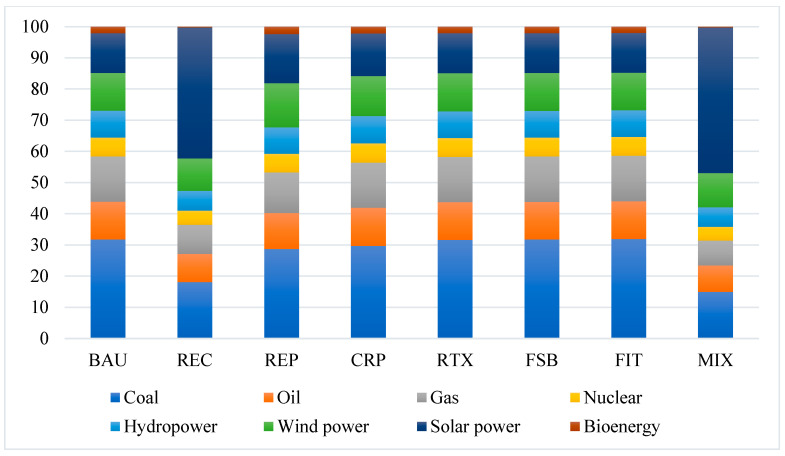
The structure of primary energy consumption in 2060 (%). Source: CHINAGEM model simulation.

Renewable energy policies will significantly raise the proportion of renewable energy in primary energy consumption (Figure 9) while increasing primary energy consumption (Table 2). Under the baseline scenario, the proportion of renewable energy in primary energy consumption will reach a level of 35.53% in 2060, which is only several percentage points higher than that in 2020. Primary energy consumption in 2060 will reach a level of 6294.34 Mtce (million tons of standard coal equivalent). If the renewable energy policies are implemented together (MIX), the proportion of renewable energy will increase to 64.19%, which is almost four times the level in 2020. Solar and wind power will account for 46.72% and 10.96% of primary energy consumption, respectively. Meanwhile, fossil fuel will still account for approximately 30% of primary energy consumption. Under the MIX scenario, primary energy consumption in 2060 will increase to 8615.96 Mtce, 36.88% higher than that under the baseline scenario. The increase in the proportion of renewable energy in primary energy consumption is dominantly driven by the REC policy, which will raise the proportion of renewable energy by 23.47 percentage points. The increase in primary energy consumption is also mostly attributed to the REC policy, as it reduces the generation cost of renewable energy and lowers down electricity price, which consequently stimulates sectors’ production and energy consumption. While the CRP, RTX, FSB, and FIT policy will reduce primary energy consumption slightly through raising the cost of fossil-fuel consumption, the REC and REP will increase primary energy consumption to different degrees.

## 4. Discussion

Renewable energy policies could play a significant role in achieving China’s dual decarbonization goals. There exists a large disparity in previous studies on the effectiveness of renewable energy policies in abating CO_2_ emissions. Several recent studies have suggested that renewable energy policies cannot effectively abate CO_2_ emissions. Different from these studies, we found that the current renewable energy policies could mitigate CO_2_ emissions by 2.57 billion tons in 2060, accounting for 30.59% of China’s carbon emissions under the baseline scenario. The proportion of renewable energy will increase to 64.19%, almost four times that in 2020. Hence, the current policies could play a significant role in achieving China’s dual decarbonization goals. However, renewable policies have greater effectiveness in promoting the substitution of renewable energy for fossil-fired electricity, rather than fossil fuels. Firms and residents more easily switch their power sources from fossil-fired electricity to renewable energy. However, the substitution of renewable energy for fossil fuels is much more costly and technically difficult, as it requires a great amount of investment in electrification reconstruction. Hence, renewable energy policy could play a vital role in accelerating China’s low-carbon transformation and achieving the dual decarbonization goals.

The optimal choices of renewable energy policies are very different depending on the selection of criteria. Renewable energy policies have very different impacts on the GDP and CO_2_ emissions. The REC policy is the only one that will abate CO_2_ emissions effectively and benefit the GDP. While the REP and CRP policies could abate CO_2_ emissions by over 0.5 billion tons CO_2_, they will also cause greater damage to the GDP, compared with the RTX and FSB policies. If policymakers attach more importance to the effectiveness in abating CO_2_ emissions, REC policies should be given more priority, followed by REP and CRP policies. If policymakers aim to reduce the GDP losses of carbon emission abatement, they should adopt the REC, RTX, and FSB policies. However, according to the efficiency index, the REC policy is the optimal choice, and the REP and CRP policies are the second-best choices. The FIT policy is the least optimal choice according to either criterion. Therefore, the optimal choices of renewable energy policies are highly correlated with the criteria. As China’s government tends to abate CO_2_ emissions at a relatively low cost, the REC, REP, and CRP policies are more suitable for achieving its dual decarbonization goals.

The technological progress in renewable energy is vital in accelerating the development of renewable energy and abating CO_2_ emissions. In 2060, the REC policy will reduce China’s CO_2_ emissions in 2060 by 1.636 billion tons and increase the GDP in 2020–2060 by accumulatively 1.1713%. It also raises the proportion of renewable energy in primary energy consumption in 2060 to 59.00%. The economic benefits of renewable energy policies on the economy and energy transformation are dominantly derived from the REC policy. Hence, the continuous technological progress in renewable energy is vital to achieve the dual decarbonization goals. China also has great potential for developing renewable energy, considering its abundant unexploitable endowments, ambitious investment, and generous supportive policies. China’s technologically exploitable wind power and solar power are estimated to be 3500 GW and 2200 GW, respectively. Less than 10% of the technologically exploitable renewable energy has been utilized by 2021. China persistently raised the investment in the development renewable energy in the past decade. In 2019, China pumped 83.4 billion U.S. dollars into the research and development, infrastructure construction, and pilot programs related to renewable energy, around 38% of global renewable energy investment. During the 14th Five Year Plan Period (2021–2025), the research and development expenditure on renewable energy will increase by over annually 7%. China’s government also enforced a series of supportive policies to encourage the technological progress and applications and increase the proportion of renewable energy, with an ambitious target that renewable energy will become the main power source by 2035. However, the rapid development of renewable energy may pose challenges to the stable operation of power grids, owing to the intermittence of wind and solar power, which could be alleviated by the installation of power storage facilities, which could be alleviated by the installation of power storage facilities.

Considering the residual CO_2_ emissions in 2060, more ambitious carbon emission abatement policies are needed. The current renewable energy policies will abate CO_2_ emissions in 2020 by 2.57 billion tons, which indicates that the residual CO_2_ emissions in 2060 will be 5.72 billion tons. Considering the carbon sink of natural ecosystems and carbon capture, utilization, and storage (CCUS), China’s residual CO_2_ emissions in 2060 should be reduced to below 2 billion tons to achieve the carbon neutrality goal. Hence, the current renewable energy policies could not achieve the carbon neutrality goal. More ambitious carbon emission abatement policies are needed. The high carbon price and the maintenance of feed-in tariffs could stimulate firms and residents to reduce their combustion of fossil fuels and accompanying carbon emissions. A powerful policy should be adopted to improve the energy efficiency of firms and residents and raise the electrification rate to reduce their consumption of fossil fuels. A deep economic transformation is also needed, cutting down the proportion of energy-intensive industries and improving the total factor productivity of firms.

This study still has several limitations. First, the means for recycling the carbon tax revenue may change the impact of the carbon market. Previous studies have developed separate ways for recycling carbon tax revenue as a lump-sum tax, the transfer to residents, the transfer to firms, or a new investment. This study assumes that the carbon tax revenue will be returned to the producers, which may lead to a result different from previous studies. Second, there is great uncertainty regarding renewable energy policies in the coming decades. This study analyzes the effects of the current renewable energy policies, as it is much more difficult to predict future policies. The governmental choices of renewable energy policies may generate uncertainty in our simulation results. Third, the CHINAGEM model, as a typical dynamic CGE model, has not explicitly considered the resource constraints for developing renewable energy, such as fresh water and rare materials. The development of solar power in dry inland areas is restricted by the availability of fresh water. The large-scale production of photovoltaic panels and wind turbines is still challenged by the deficiency of rare materials, including neodymium, praseodymium, and dysprosium.

## 5. Conclusions

To achieve ambitious dual decarbonization goals, China’s government has implemented a series of policies to accelerate the development of renewable energy. However, the effectiveness and trade-offs of renewable energy policies in achieving the dual decarbonization goals are poorly understood in previous studies. This study employs a dynamic CGE model to evaluate the impact of renewable energy policies on China’s future carbon emissions and economy, and reveals the effectiveness and trade-offs of renewable energy policies in achieving the dual decarbonization goals. This study contributes to the existing literature in the following ways. First, the effectiveness of renewable energy policies in abating China’s carbon emissions toward 2060 are quantitively evaluated. Second, the trade-offs of renewable energy policies are investigated, which reveals the costs of these policies more comprehensively. Third, an efficiency index for different renewable energy policies is created to describe the suitability of these policies in achieving China’s dual decarbonization goals.

The simulation results of this study suggest that first, except for reducing feed-in tariffs (FITs), renewable energy policies could reduce China’s CO_2_ emissions in 2060, but their effectiveness in abating CO_2_ emissions is very different. The reduction in renewable energy cost (REC) would have the greatest effectiveness in abating CO_2_ emissions toward 2060, reducing 1.636 billion tons CO_2_ in 2060, followed by the policies of renewable energy portfolio standard (REP) and carbon market (CRP). If renewable policies are implemented together (MIX), CO_2_ emissions in 2060 will decrease by 2.57 billion tons, accounting for 30.59% of China’s carbon emissions under the baseline scenario. Second, if measuring the abatement cost of renewable energy policies by the changes in China’s GDP, renewable energy policies would cause relatively slight damage to China’s GDP, with the only exception of the REC. The CRP policy would cause the largest economic loss, by reducing China’s GDP by accumulatively 0.0989% in 2020–2060, followed by the REP and RTX policies. Compared with these policies, the negative impacts of the FSB and FIT policies are much softer, reducing the GDP by accumulatively 0.0023% and 0.0019%, respectively, in 2020–2060. The REC policy could benefit the economic growth significantly by raising China’s GDP by accumulatively 1.1713% in 2020–2060, which generates the positive economic impact of the policy portfolio. Third, regarding the trade-offs of renewable energy policies, most of them will sacrifice China’s internal demand (i.e., investment and consumption), but benefit the employment. They would also cause damage to the external demand, except for the REC policy. Only the REC policy could benefit the internal and external demand simultaneously, while abating the CO_2_ emissions most effectively. Fourth, by ranking renewable energy policies according to the efficiency indicator, the REC policy is the most suitable policy to promote the development of renewable energy, followed by the REP and CRP. The RTX and FSB policies are of the least suitability for achieving China’s dual decarbonization goals. Fifth, renewable energy policies will deeply reshape the structure of energy production in China by reducing the output of fossil fuels and significantly increasing the proportion of renewable energy in electricity generation. The proportion of renewable energy in primary energy consumption will increase significantly, transferring the energy structure to a low carbon one.

## Figures and Tables

**Table 1:** The accumulative impacts of renewable energy policies on macroeconomic indicators in 2020–2060 relative to the baseline scenario (%).

	REC	REP	CRP	RTX	FSB	FIT	MIX
GDP	1.1713	−0.0384	−0.0989	−0.0144	−0.0023	−0.0019	1.1811
Employment	0.0936	0.0416	0.0065	0.0045	0.0009	−0.0005	0.1720
Investment	0.4040	0.2760	−0.0533	−0.0040	−0.0011	−0.0031	0.6384
Consumption	1.1704	−0.0487	−0.0638	−0.0132	−0.0019	−0.0011	1.2109
Export	0.9507	−0.1121	−0.0665	−0.0221	−0.0031	−0.0007	0.9081
Import	0.2933	−0.0679	−0.0432	−0.0130	−0.0015	0.0004	0.2356
CPI	−0.4773	−0.0755	0.0100	0.0003	−0.0003	0.0001	−0.6017
Labor price	0.9971	−0.0989	−0.0486	−0.0405	−0.0068	−0.0007	0.9429
Capital price	−1.0690	0.0988	0.2080	−0.0008	−0.0017	0.0118	−0.9137

Source: CHINAGEM model simulation.

**Table 2:** Primary energy consumption in 2060 (million tce).

	BAU	REC	REP	CRP	RTX	FSB	FIT	MIX
Primary Energy consumption	6294.34	8429.41	6365.04	6154.92	6280.68	6293.48	6290.24	8615.96

Source: CHINAGEM model simulation.

## Data Availability

Not applicable.

## Appendix A

**Table A1:** The summary of renewable energy policies.

Policies	Abbr.	Scenario Design
Carbon market	CPR	Carbon price in China will increase from 100 RMB/ton CO_2_ in 2020 to 100 USD/ton CO_2_ in 2060
Reduction of feed-in tariff	FIT	Feed-in tariffs on fossil-fuel fired powers and subsidies on renewable energy will be canceled from 2020
Reduction in fossil fuel subsidies	FBS	Governmental subsidies on fossil fuel will be reduced from 2020
Reduction of renewable energy cost	REC	Generation costs of renewable powers would decline by an annual average of 0.505% to 2.954% in 2020–2060
Resource tax	RTX	Resource tax rates on coal, crude oil, and crude gas will increase from 6% to 7.5% in 2020
Renewable energy portfolio standard	REP	Proportion of renewable energy in primary energy consumption will increase by an additional 5 percent in 2020–2060
Mix of renewable energy policies	Mix	The aforementioned policies will be implemented simultaneously
